# The genome sequence of the Common White Wave, *Cabera pusaria *(Linnaeus, 1758)

**DOI:** 10.12688/wellcomeopenres.20135.1

**Published:** 2023-10-13

**Authors:** Denise C. Wawman, University of Oxford and Wytham Woods Genome Acquisition Lab, Darwin Tree of Life Barcoding collective, Wellcome Sanger Institute Tree of Life programme, Wellcome Sanger Institute Scientific Operations: DNA Pipelines collective, Tree of Life Core Informatics collective, Darwin Tree of Life Consortium

**Affiliations:** 1 Edward Grey Institute, Department of Biology, University of Oxford, Oxford, England, UK

**Keywords:** Cabera pusaria, common white wave, genome sequence, chromosomal, Lepidoptera

## Abstract

We present a genome assembly from an individual male *Cabera pusaria* (the Common White Wave; Arthropoda; Insecta; Lepidoptera; Geometridae). The genome sequence is 794.3 megabases in span. Most of the assembly is scaffolded into 31 chromosomal pseudomolecules, including the Z sex chromosome. The mitochondrial genome has also been assembled and is 15.64 kilobases in length.

## Species taxonomy

Eukaryota; Metazoa; Eumetazoa; Bilateria; Protostomia; Ecdysozoa; Panarthropoda; Arthropoda; Mandibulata; Pancrustacea; Hexapoda; Insecta; Dicondylia; Pterygota; Neoptera; Endopterygota; Amphiesmenoptera; Lepidoptera; Glossata; Neolepidoptera; Heteroneura; Ditrysia; Obtectomera; Geometroidea; Geometridae; Ennominae; *Cabera*; *Cabera pusaria* (Linnaeus, 1758) (NCBI:txid722659).

## Background

*Cabera pusaria* is a moth in the family Geometridae, the second largest family of macro-moths in the British Isles, and like most of the members of this family has a thin body and triangular wings ( Waring & Townsend, 2017). It has white wings with grey cross-lines, and can be distinguished from the similar Common Wave *Cabera exanthemata* by the straightness of the outer cross-lines towards the leading edge of the wing, compared to the curved lines in *C. exanthemata* ( Skinner, 2009; Waring & Townsend, 2017).

*C. pusaria* is found throughout the British Isles where it is often disturbed from vegetation during the day and comes to light in small numbers, flying between late-May and early-September in the south where it is double-brooded and from May to July in the north where it is single-brooded. The larvae feed on a range of trees – including Downy Birch *Betula pubescens*, Silver Birch *Betula pendula*, Sallow *Salix* spp., Alder *Alnus* spp., Oak *Quercus* spp., and Sweet Chestnut *Castanea sativa* - from July to September, before pupating to overwinter ( Skinner, 2009; Waring & Townsend, 2017).

While trace elements are important in the diet, high doses of the heavy metal manganese are fatal to the larvae and pupae of *C. pusaria* ( Martinek *et al.*, 2020), however, the larvae were the most numerous species on Birch *Betula pendula* in an area of the Czech Republic polluted by sulphur dioxide where they are considered pests ( Kula *et al.*, 2005).

We present a chromosomally complete genome sequence for *Cabera pusaria*, based on one male specimen collected using a mercury vapour light trap in a rural garden in the hamlet of Bratton, near Minehead, in Somerset, as part of the Darwin Tree of Life Project. This project is a collaborative effort to sequence all named eukaryotic species in the Atlantic Archipelago of Britain and Ireland.

## Genome sequence report

**Figure 1.**
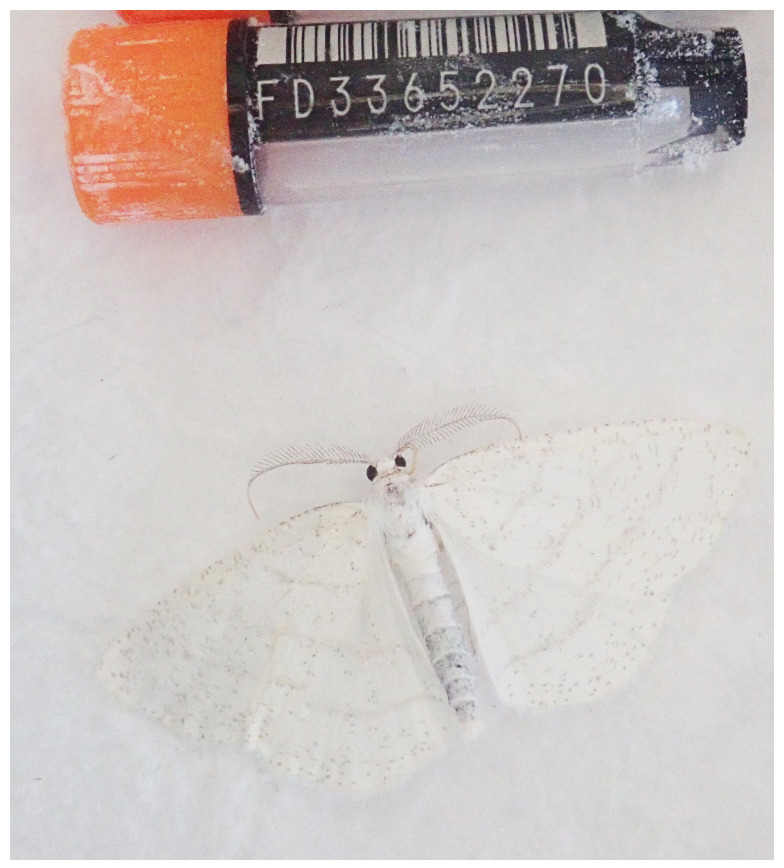
Photograph of the *Cabera pusaria* (ilCabPusa1) specimen used for genome sequencing.

The genome was sequenced from one male *Cabera pusaria* ( Figure 1) collected from Bratton, Somerset, UK (51.20, –3.51). A total of 27-fold coverage in Pacific Biosciences single-molecule HiFi long reads was generated. Primary assembly contigs were scaffolded with chromosome conformation Hi-C data. Manual assembly curation corrected 30 missing joins or mis-joins, reducing the assembly length by 0.75% and the scaffold number by 5.08%.

**Figure 2.**
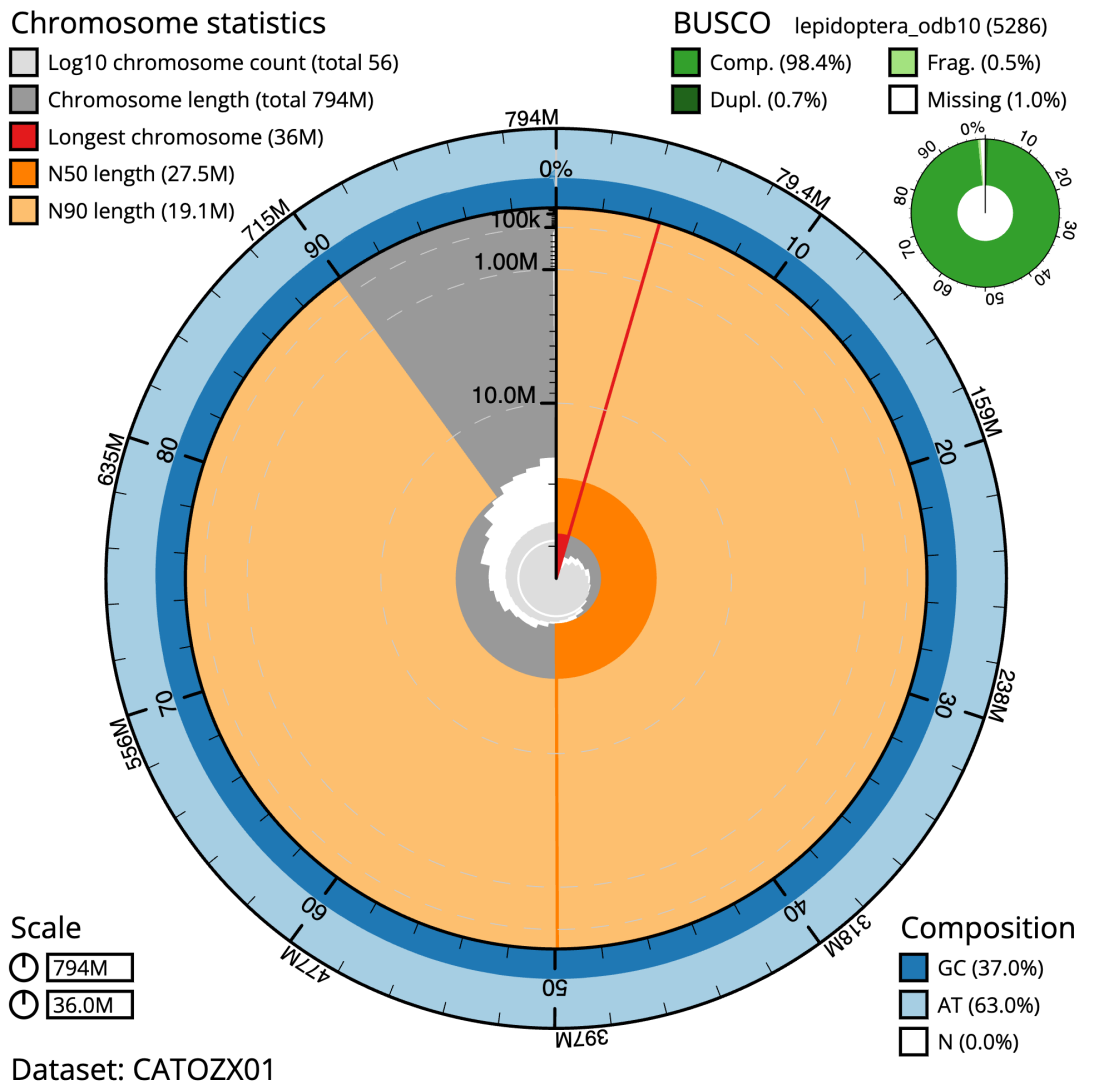
Genome assembly of *Cabera pusaria*, ilCabPusa1.1: metrics. The BlobToolKit Snailplot shows N50 metrics and BUSCO gene completeness. The main plot is divided into 1,000 size-ordered bins around the circumference with each bin representing 0.1% of the 794,294,107 bp assembly. The distribution of scaffold lengths is shown in dark grey with the plot radius scaled to the longest scaffold present in the assembly (35,978,249 bp, shown in red). Orange and pale-orange arcs show the N50 and N90 scaffold lengths (27,510,719 and 19,118,028 bp), respectively. The pale grey spiral shows the cumulative scaffold count on a log scale with white scale lines showing successive orders of magnitude. The blue and pale-blue area around the outside of the plot shows the distribution of GC, AT and N percentages in the same bins as the inner plot. A summary of complete, fragmented, duplicated and missing BUSCO genes in the lepidoptera_odb10 set is shown in the top right. An interactive version of this figure is available at https://blobtoolkit.genomehubs.org/view/ilCabPusa1.1/dataset/CATOZX01/snail.

**Figure 3.**
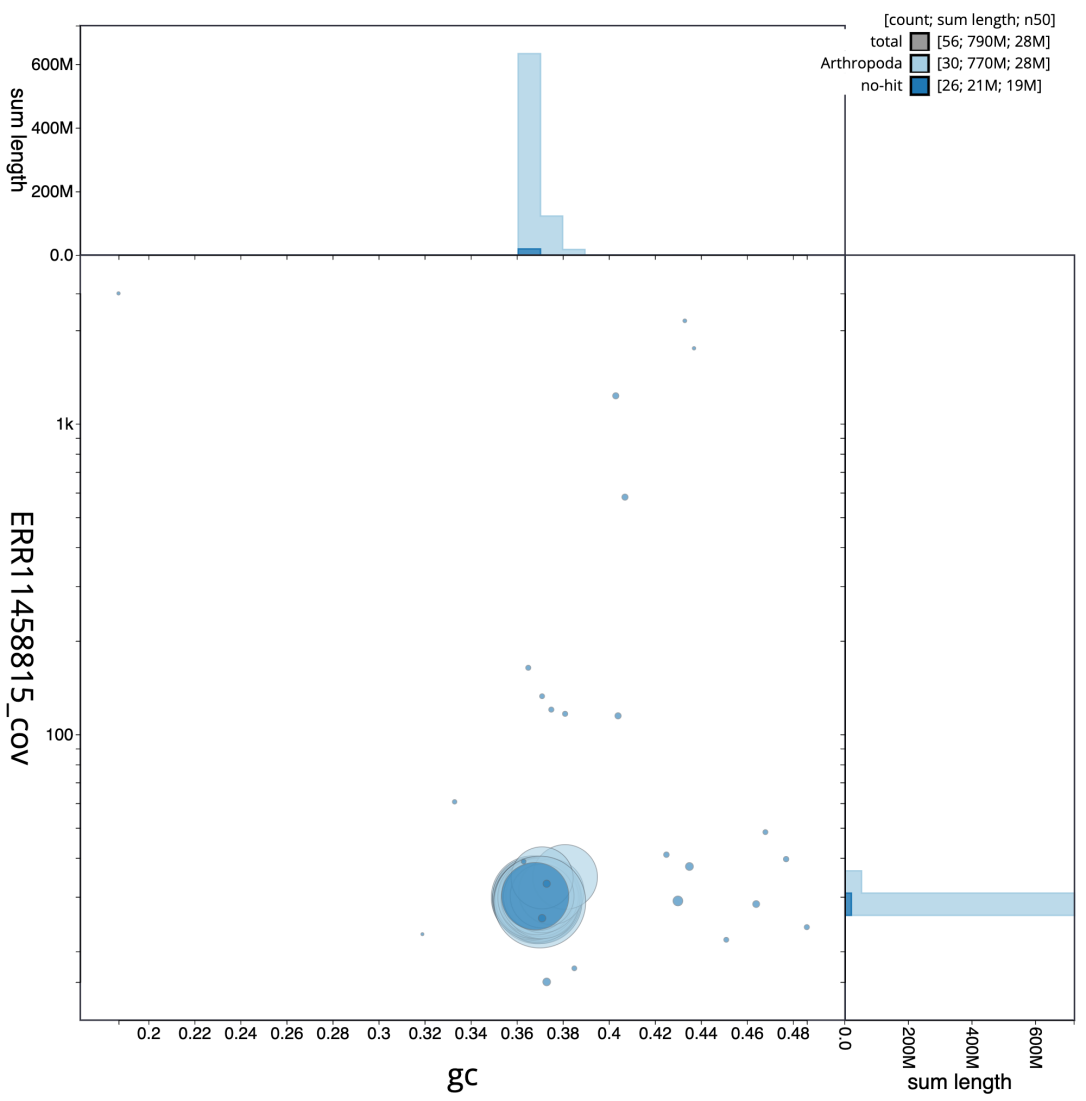
Genome assembly of *Cabera pusaria*, ilCabPusa1.1: BlobToolKit GC-coverage plot. Scaffolds are coloured by phylum. Circles are sized in proportion to scaffold length. Histograms show the distribution of scaffold length sum along each axis. An interactive version of this figure is available at https://blobtoolkit.genomehubs.org/view/ilCabPusa1.1/dataset/CATOZX01/blob.

**Figure 4.**
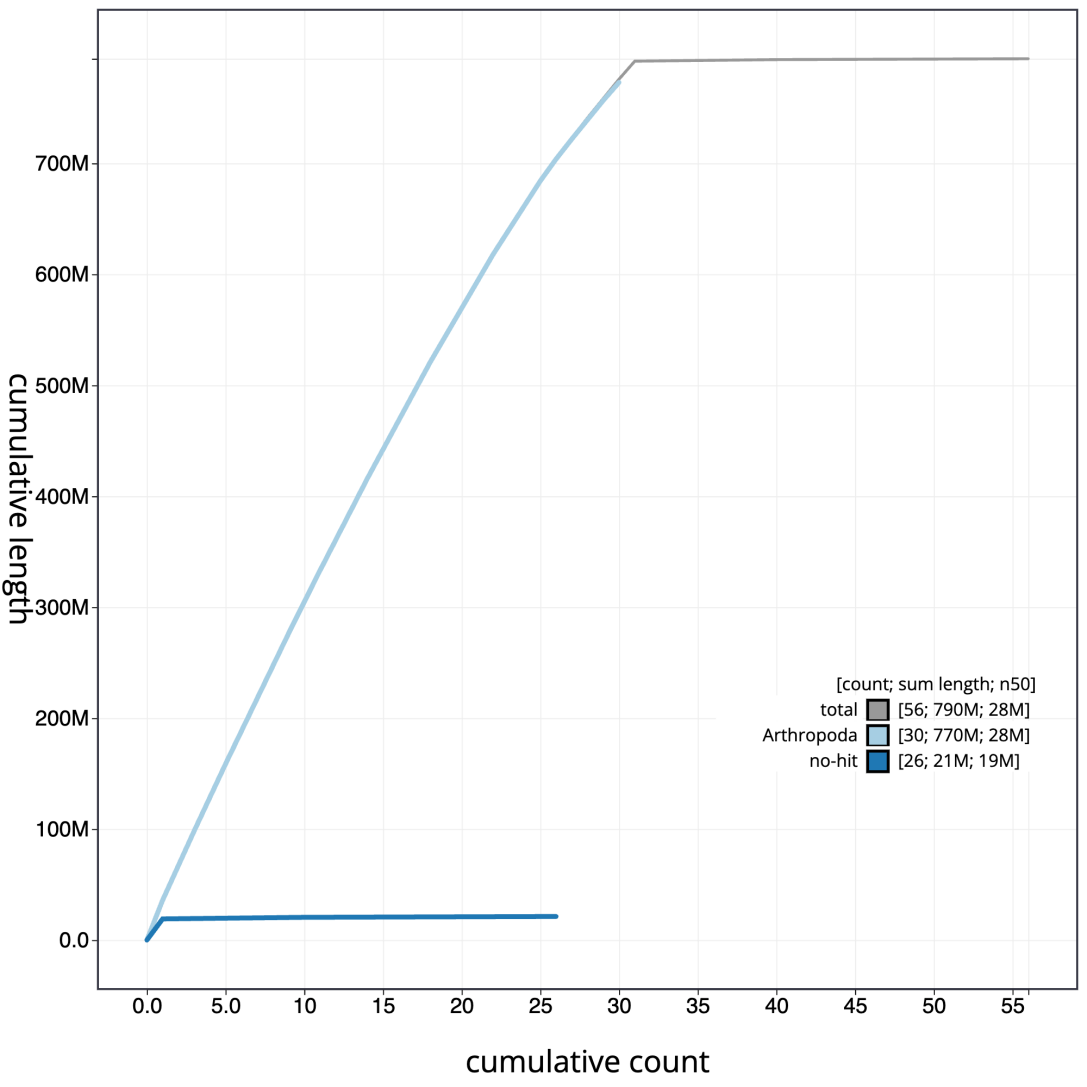
Genome assembly of *Cabera pusaria*, ilCabPusa1.1: BlobToolKit cumulative sequence plot. The grey line shows cumulative length for all scaffolds. Coloured lines show cumulative lengths of scaffolds assigned to each phylum using the buscogenes taxrule. An interactive version of this figure is available at https://blobtoolkit.genomehubs.org/view/ilCabPusa1.1/dataset/CATOZX01/cumulative.

**Figure 5.**
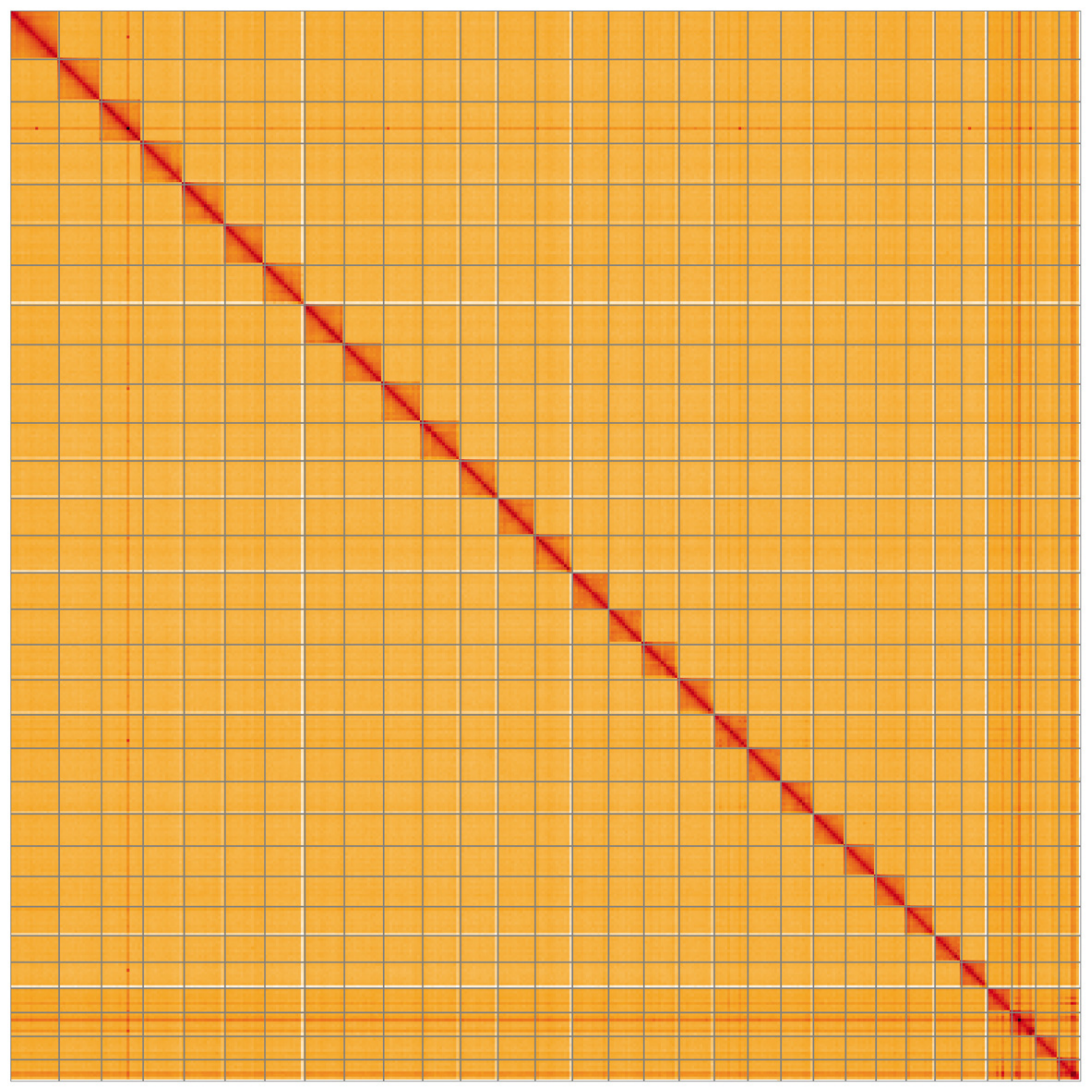
Genome assembly of *Cabera pusaria*, ilCabPusa1.1: Hi-C contact map of the ilCabPusa1.1 assembly, visualised using HiGlass. Chromosomes are shown in order of size from left to right and top to bottom. An interactive version of this figure may be viewed at https://genome-note-higlass.tol.sanger.ac.uk/l/?d=ZJRZI5v_TS6R5ve-N46dqA.

The final assembly has a total length of 794.3 Mb in 55 sequence scaffolds with a scaffold N50 of 27.5 Mb ( Table 1). The snailplot in Figure 2 provides a summary of the assembly statistics, while the distribution of assembly scaffolds on GC proportion and coverage is shown in Figure 3. The cumulative assembly plot in Figure 4 shows curves for subsets of scaffolds assigned to different phyla. Most (99.72%) of the assembly sequence was assigned to 31 chromosomal-level scaffolds, representing 30 autosomes and the Z sex chromosome. Chromosome Z was assigned based on synteny to *Petrophora chlorosata* (GCA_951640565.1). Chromosome-scale scaffolds confirmed by the Hi-C data are named in order of size ( Figure 5; Table 2). While not fully phased, the assembly deposited is of one haplotype. Contigs corresponding to the second haplotype have also been deposited. The mitochondrial genome was also assembled and can be found as a contig within the multifasta file of the genome submission.

**Table 1.** Genome data for *Cabera pusaria*, ilCabPusa1.1.

Project accession data		
Assembly identifier	ilCabPusa1.1	
Assembly release date	2023-07-14	
Species	*Cabera pusaria*	
Specimen	ilCabPusa1	
NCBI taxonomy ID	722659	
BioProject	PRJEB62171	
BioSample ID	SAMEA112226456	
Isolate information	ilCabPusa1, male: head and thorax (DNA) ilCabPusa2, male: head (Hi-C and RNA sequencing)	
Assembly metrics *		*Benchmark*
Consensus quality (QV)	62.8	*≥ 50*
*k*-mer completeness	100%	*≥ 95%*
BUSCO **	C:98.4%[S:97.8%,D:0.7%],F:0.5%, M:1.0%,n:5,286	*C ≥ 95%*
Percentage of assembly mapped to chromosomes	99.72%	*≥ 95%*
Sex chromosomes	Z chromosome	*localised homologous pairs*
Organelles	Mitochondrial genome assembled	*complete single alleles*
Raw data accessions		
PacificBiosciences SEQUEL II	ERR11458815	
Hi-C Illumina	ERR11468744	
PolyA RNA-Seq Illumina	ERR11468743	
Genome assembly		
Assembly accession	GCA_954871355.1	
*Accession of alternate haplotype*	GCA_954871345.1	
Span (Mb)	794.3	
Number of contigs	178	
Contig N50 length (Mb)	7.6	
Number of scaffolds	55	
Scaffold N50 length (Mb)	27.5	
Longest scaffold (Mb)	36.0	

* Assembly metric benchmarks are adapted from column VGP-2020 of “Table 1: Proposed standards and metrics for defining genome assembly quality” from ( Rhie *et al.*, 2021). ** BUSCO scores based on the lepidoptera_odb10 BUSCO set using v5.3.2. C = complete [S = single copy, D = duplicated], F = fragmented, M = missing, n = number of orthologues in comparison. A full set of BUSCO scores is available at https://blobtoolkit.genomehubs.org/view/ilCabPusa1.1/dataset/CATOZX01/busco.

**Table 2.** Chromosomal pseudomolecules in the genome assembly of *Cabera pusaria*, ilCabPusa1.

INSDC accession	Chromosome	Length (Mb)	GC%
OX940886.1	1	31.52	37.0
OX940887.1	2	30.76	37.0
OX940888.1	3	30.33	37.0
OX940889.1	4	30.22	37.0
OX940890.1	5	29.56	37.0
OX940891.1	6	29.51	36.5
OX940892.1	7	29.3	37.0
OX940893.1	8	29.12	37.0
OX940894.1	9	28.75	37.0
OX940895.1	10	28.07	36.5
OX940896.1	11	27.64	36.5
OX940897.1	12	27.55	37.0
OX940898.1	13	27.51	37.0
OX940899.1	14	26.9	37.0
OX940900.1	15	26.15	36.5
OX940901.1	16	26.11	37.0
OX940902.1	17	25.82	37.0
OX940903.1	18	24.91	37.0
OX940904.1	19	24.52	37.0
OX940905.1	20	23.92	37.0
OX940906.1	21	23.88	37.0
OX940907.1	22	22.41	37.0
OX940908.1	23	22.31	37.0
OX940909.1	24	21.47	37.0
OX940910.1	25	19.65	37.0
OX940911.1	26	19.12	37.0
OX940912.1	27	18.08	37.5
OX940913.1	28	17.64	38.0
OX940914.1	29	17.19	37.0
OX940915.1	30	16.2	37.0
OX940885.1	Z	35.98	37.0
OX940916.1	MT	0.02	19.0

The estimated Quality Value (QV) of the final assembly is 62.8 with *k*-mer completeness of 100%, and the assembly has a BUSCO v5.3.2 completeness of 98.4% (single = 97.8%, duplicated = 0.7%), using the lepidoptera_odb10 reference set ( *n* = 5,286).

Metadata for specimens, spectral estimates, sequencing runs, contaminants and pre-curation assembly statistics can be found at https://tolqc.cog.sanger.ac.uk/darwin/insects/Cabera_pusaria/.

## Methods

### Sample acquisition and nucleic acid extraction

The specimens used in this study were collected in a light trap in a garden in Bratton, Somerset, UK (latitude 51.20, longitude –3.51) on 2022-06-20. The specimens were collected and identified by Denise Wawman (University of Oxford) and were snap-frozen on dry ice. The specimen with ID Ox002230 (ToLID ilCabPusa1) was used for DNA sequencing, while the specimen with ID Ox002236 (ToLID ilCabPusa2) was used for Hi-C analysis and RNA sequencing.

The ilCabPusa1 sample was prepared for DNA extraction at the Tree of Life laboratory, Wellcome Sanger Institute (WSI). The specimen was weighed and dissected on dry ice with tissue set aside for Hi-C sequencing. Head and thorax tissue was disrupted using a Nippi Powermasher fitted with a BioMasher pestle. DNA was extracted at the WSI Scientific Operations core using the Qiagen MagAttract HMW DNA kit, according to the manufacturer’s instructions.

RNA was extracted from head tissue of ilCabPusa2 in the Tree of Life Laboratory at the WSI using TRIzol, according to the manufacturer’s instructions. RNA was then eluted in 50 μl RNAse-free water and its concentration assessed using a Nanodrop spectrophotometer and Qubit Fluorometer using the Qubit RNA Broad-Range (BR) Assay kit. Analysis of the integrity of the RNA was done using Agilent RNA 6000 Pico Kit and Eukaryotic Total RNA assay.

### Sequencing

Pacific Biosciences HiFi circular consensus DNA sequencing libraries were constructed according to the manufacturers’ instructions. Poly(A) RNA-Seq libraries were constructed using the NEB Ultra II RNA Library Prep kit. DNA and RNA sequencing was performed by the Scientific Operations core at the WSI on Pacific Biosciences SEQUEL II (HiFi) and Illumina NovaSeq 6000 (RNA-Seq) instruments. Hi-C data were also generated from head tissue of ilCabPusa2 using the Arima2 kit and sequenced on the Illumina NovaSeq 6000 instrument.

### Genome assembly, curation and evaluation

Assembly was carried out with Hifiasm ( Cheng *et al.*, 2021) and haplotypic duplication was identified and removed with purge_dups ( Guan *et al.*, 2020). The assembly was then scaffolded with Hi-C data ( Rao *et al.*, 2014) using YaHS ( Zhou *et al.*, 2023). The assembly was checked for contamination and corrected as described previously ( Howe *et al.*, 2021). Manual curation was performed using HiGlass ( Kerpedjiev *et al.*, 2018) and Pretext ( Harry, 2022). The mitochondrial genome was assembled using MitoHiFi ( Uliano-Silva *et al.*, 2023), which runs MitoFinder ( Allio *et al.*, 2020) or MITOS ( Bernt *et al.*, 2013) and uses these annotations to select the final mitochondrial contig and to ensure the general quality of the sequence.

A Hi-C map for the final assembly was produced using bwa-mem2 ( Vasimuddin *et al.*, 2019) in the Cooler file format ( Abdennur & Mirny, 2020). To assess the assembly metrics, the *k*-mer completeness and QV consensus quality values were calculated in Merqury ( Rhie *et al.*, 2020). This work was done using Nextflow ( Di Tommaso *et al.*, 2017) DSL2 pipelines “sanger-tol/readmapping” ( Surana *et al.*, 2023a) and “sanger-tol/genomenote” ( Surana *et al.*, 2023b). The genome was analysed within the BlobToolKit environment ( Challis *et al.*, 2020) and BUSCO scores ( Manni *et al.*, 2021; Simão *et al.*, 2015) were calculated.

Table 3 contains a list of relevant software tool versions and sources.

**Table 3.** Software tools: versions and sources.

Software tool	Version	Source
BlobToolKit	4.1.7	https://github.com/blobtoolkit/blobtoolkit
BUSCO	5.3.2	https://gitlab.com/ezlab/busco
Hifiasm	0.16.1-r375	https://github.com/chhylp123/hifiasm
HiGlass	1.11.6	https://github.com/higlass/higlass
Merqury	MerquryFK	https://github.com/thegenemyers/MERQURY.FK
MitoHiFi	3	https://github.com/marcelauliano/MitoHiFi
PretextView	0.2	https://github.com/wtsi-hpag/PretextView
purge_dups	1.2.5	https://github.com/dfguan/purge_dups
sanger-tol/genomenote	v1.0	https://github.com/sanger-tol/genomenote
sanger-tol/readmapping	1.1.0	https://github.com/sanger-tol/readmapping/tree/1.1.0
YaHS	1.2a.2	https://github.com/c-zhou/yahs

### Wellcome Sanger Institute – Legal and Governance

The materials that have contributed to this genome note have been supplied by a Darwin Tree of Life Partner. The submission of materials by a Darwin Tree of Life Partner is subject to the **‘Darwin Tree of Life Project Sampling Code of Practice’**, which can be found in full on the Darwin Tree of Life website here. By agreeing with and signing up to the Sampling Code of Practice, the Darwin Tree of Life Partner agrees they will meet the legal and ethical requirements and standards set out within this document in respect of all samples acquired for, and supplied to, the Darwin Tree of Life Project.

Further, the Wellcome Sanger Institute employs a process whereby due diligence is carried out proportionate to the nature of the materials themselves, and the circumstances under which they have been/are to be collected and provided for use. The purpose of this is to address and mitigate any potential legal and/or ethical implications of receipt and use of the materials as part of the research project, and to ensure that in doing so we align with best practice wherever possible. The overarching areas of consideration are:

•   Ethical review of provenance and sourcing of the material

•   Legality of collection, transfer and use (national and international)

Each transfer of samples is further undertaken according to a Research Collaboration Agreement or Material Transfer Agreement entered into by the Darwin Tree of Life Partner, Genome Research Limited (operating as the Wellcome Sanger Institute), and in some circumstances other Darwin Tree of Life collaborators.

## Data Availability

European Nucleotide Archive: *Cabera pusaria* (common white wave). Accession number PRJEB62171; https://identifiers.org/ena.embl/PRJEB62171. ( Wellcome Sanger Institute, 2023) The genome sequence is released openly for reuse. The *Cabera pusaria* genome sequencing initiative is part of the Darwin Tree of Life (DToL) project. All raw sequence data and the assembly have been deposited in INSDC databases. The genome will be annotated using available RNA-Seq data and presented through the Ensembl pipeline at the European Bioinformatics Institute. Raw data and assembly accession identifiers are reported in Table 1.

## Author information

Members of the University of Oxford and Wytham Woods Genome Acquisition Lab are listed here: https://doi.org/10.5281/zenodo.4789928.

Members of the Darwin Tree of Life Barcoding collective are listed here: https://doi.org/10.5281/zenodo.4893703.

Members of the Wellcome Sanger Institute Tree of Life programme are listed here: https://doi.org/10.5281/zenodo.4783585.

Members of Wellcome Sanger Institute Scientific Operations: DNA Pipelines collective are listed here: https://doi.org/10.5281/zenodo.4790455.

Members of the Tree of Life Core Informatics collective are listed here: https://doi.org/10.5281/zenodo.5013541.

Members of the Darwin Tree of Life Consortium are listed here: https://doi.org/10.5281/zenodo.4783558.
