# Supplementary material for: The genome sequence of the Common White Wave, Cabera pusaria (Linnaeus, 1758)
Source: Wellcome Open Res. 2023 Oct 13;8:460. [Version 1] doi: 10.12688/wellcomeopenres.20135.1 (PMC11167327; doi:10.12688/wellcomeopenres.20135.1)
Supplement: Peer review history

# Reviewer report: Reviewer response for version 1

**DOI:** 10.21956/wellcomeopenres.22297.r84179

**Published:** 2024-06-11

**Authors:** Frederique Hilliou

**Affiliations:** 1 INRAE, Institut Sophia Agrobiotech, Universite Cote d'Azur, Nice, Provence-Alpes-Côte d'Azur, France

The manuscript describes the obtention of the genome of *Cabera pusaria* at the chromosome level.

 The genome of *C pusaria* could be used for the study of lepidopteran adaptation to heavy metal. Genomic comparison of the populations showing different level of tolerance to heavy metal could also be performed. Martinek et al 2020 [Ref- 1] described a population of *C pusaria* able to eliminate excess of the heavy metal manganese. More genomic formation are needed to understand how insect populations adapt to polluted environment (Kozlov,2022) [Ref- 2]

 A complete genome publication should propose a gene annotation that is still missing here. I suggest to update the article with this data.

 Once, gene annotations will be available, it will be possible to determine which detoxification pathway are involved in the excretion of manganese.  What genome modification have led to the capacities of some *C pusaria* populations to survive on high concentration of heavy metals? Are these genomic regions already involved in adaptation of lepidopteran to anthropisation of the environment?

 I have other questions that could help understand the data presented in this manuscript.

 Could you explain why some "no hit data" in figure3 have the same GC contents as *C pusaria*? How could you differentiate these scaffolds from the *C pusaria* genome scaffold?

 Where you able to find DNA from bacteria, fungi or viruses that could also give information of this insect physiology? Could you give the main origine of these contaminants.

Are sufficient details of methods and materials provided to allow replication by others?

Partly

Is the rationale for creating the dataset(s) clearly described?

Yes

Are the datasets clearly presented in a useable and accessible format?

Yes

Are the protocols appropriate and is the work technically sound?

Yes

Reviewer Expertise:

insect genomic, detoxification, cytochrome P450, comparative genomic in lepidoptera, plant insect interactions

I confirm that I have read this submission and believe that I have an appropriate level of expertise to confirm that it is of an acceptable scientific standard.

## References

[1] : Adverse responses ofCabera pusaria caterpillars to high dietary manganese concentration. *Entomologia Experimentalis et Applicata* .2020;168(8) : 10.1111/eea.12919 635-643 10.1111/eea.12919

[2] : Population dynamics of herbivorous insects in polluted landscapes. *Curr Opin Insect Sci* .2022;54: 10.1016/j.cois.2022.100987 100987 10.1016/j.cois.2022.100987 36307065

# Reviewer report: Reviewer response for version 1

**DOI:** 10.21956/wellcomeopenres.22297.r79544

**Published:** 2024-05-02

**Authors:** Suriya Narayanan Murugesan

**Affiliations:** 1 National University of Singapore,, Singapore, Singapore

Denise C. Wawman presents a report on the complete genome assembly of *Cabera pusaria*. The assembly was generated from a single male individual, with 99.72% of the sequences assigned to 31 chromosomes using Hi-C data. This work is a valuable addition to the growing collection of Lepidoptera genome assemblies and will be very helpful to the community.

 I have only a few minor suggestions:

 1. The author mentioned that annotation will be done using the Ensembl pipeline with the collected RNA-Seq data. It would be appreciated if the author could include the annotation information once it's ready, along with the BUSCO score obtained using protein sequences from the annotation, in the revision.

 2. The author mentioned that Chromosome Z was assigned based on synteny to *Petrophora chlorosata*. It would be helpful to include a line or two on how the synteny analysis was conducted and the level of synteny observed between the two species.

Are sufficient details of methods and materials provided to allow replication by others?

Yes

Is the rationale for creating the dataset(s) clearly described?

Yes

Are the datasets clearly presented in a useable and accessible format?

Yes

Are the protocols appropriate and is the work technically sound?

Yes

Reviewer Expertise:

Evo-Devo, Genetics

I confirm that I have read this submission and believe that I have an appropriate level of expertise to confirm that it is of an acceptable scientific standard.
